# Cerebrospinal Fluid Findings of 36 Adult Patients with Autism Spectrum Disorder

**DOI:** 10.3390/brainsci10060355

**Published:** 2020-06-08

**Authors:** Kimon Runge, Ludger Tebartz van Elst, Simon Maier, Kathrin Nickel, Dominik Denzel, Miriam Matysik, Hanna Kuzior, Tilman Robinson, Thomas Blank, Rick Dersch, Katharina Domschke, Dominique Endres

**Affiliations:** 1 Section for Experimental Neuropsychiatry, Department of Psychiatry and Psychotherapy, Medical Center - University of Freiburg, Faculty of Medicine, University of Freiburg, Freiburg im Breisgau, Germany; kimon.runge@uniklinik-freiburg.de (K.R.); simon.maier@uniklinik-freiburg.de (S.M.); kathrin.nickel@uniklinik-freiburg.de (K.N.); dominik.denzel@uniklinik-freiburg.de (D.D.); miriam.matysik@uniklinik-freiburg.de (M.M.); hanna.kuzior@uniklinik-freiburg.de (H.K.); dominique.endres@uniklinik-freiburg.de (D.E.); 2 Department of Psychiatry and Psychotherapy, Medical Center - University of Freiburg, Faculty of Medicine, University of Freiburg, Freiburg im Breisgau, Germany; katharina.domschke@uniklinik-freiburg.de; 3 Department of Neurology, Medical Center - University of Freiburg, Faculty of Medicine, University of Freiburg, Freiburg im Breisgau, Germany; tilman.robinson@uniklinik-freiburg.de (T.R.); rick.dersch@uniklinik-freiburg.de (R.D.); 4 Institute of Neuropathology, Medical Center - University of Freiburg, Faculty of Medicine, University of Freiburg, Freiburg im Breisgau, Germany; thomas.blank@uniklinik-freiburg.de; 5 Center for Basics in Neuromodulation, Faculty of Medicine, University of Freiburg, Freiburg im Breisgau, Germany

**Keywords:** Asperger syndrome, autism spectrum disorder, adults, cerebrospinal fluid, antibodies, blood–brain barrier, GAD65

## Abstract

Autism spectrum disorder (ASD) is a common neurodevelopmental disorder characterized by difficulties with social interaction, repetitive behavior, and additional features, such as special interests. Its precise etiology is unclear. Recently, immunological mechanisms, such as maternal autoantibodies/infections, have increasingly been the subject of discussion. Cerebrospinal fluid (CSF) investigations play a decisive role in the detection of immunological processes in the brain. This study therefore retrospectively analyzed the CSF findings of adult patients with ASD. CSF basic measures (white blood cell count, total protein, albumin quotient, immunoglobulin G (IgG) index, and oligoclonal bands) and various antineuronal antibody findings of 36 adult patients with ASD, who had received lumbar puncture, were compared with an earlier described mentally healthy control group of 39 patients with idiopathic intracranial hypertension. CSF protein concentrations and albumin quotients of patients with ASD were significantly higher as compared to controls (age corrected: *p* = 0.003 and *p* = 0.004, respectively); 17% of the patients with ASD showed increased albumin quotients. After correction for age and gender, the group effect for total protein remained significant (*p* = 0.041) and showed a tendency for albumin quotient (*p* = 0.079). In the CSF of two ASD patients, an intrathecal synthesis of anti-glutamate decarboxylase 65 (GAD65) antibodies was found. In total, more of the ASD patients (44%) presented abnormal findings in CSF basic diagnostics compared to controls (18%; *p* = 0.013). A subgroup of the patients with adult ASD showed indication of a blood–brain barrier dysfunction, and two patients displayed an intrathecal synthesis of anti-GAD65 antibodies; thus, the role of these antibodies in patients with ASD should be further investigated. The results of the study are limited by its retrospective and open design. The group differences in blood–brain barrier markers could be influenced by a different gender distribution between ASD patients and controls.

## 1. Introduction

Autism spectrum disorder (ASD) is a common neurodevelopmental disorder characterized by deficits in social interaction and communication, unusually narrowed interests, and repetitive behavior in different situations [1]. Furthermore, the disorder involves specific alterations in social and sensory perception as well as in language [2]. The description of ASD in the Diagnostic and Statistical Manual of Mental Disorders, version 5 (DSM-5) demonstrates the clear tendency of the scientific community to subsume the various previous autism subtypes of the DSM-IV and of the International Statistical Classification of Diseases and Related Health Problems, version 10 (ICD-10) (childhood autism (ICD-10 F84.0, corresponding in the DSM-IV to autistic disorder 299.00), atypical autism (ICD-10 F84.1, not listed in the DSM-IV), and Asperger syndrome (ICD-10 F84.5, DSM-IV 299.80)) under a single diagnostic category [1,3,4]. ASD is a lifelong condition affecting predominantly males that is already present in early childhood, persists into adulthood, and is often accompanied by a variety of psychiatric comorbidities [1,2]. Despite the growing public and scientific interest in ASD over the past decades [5], which reflects increasing prevalence rates that are well above 1% and up to 2.7% depending on the populations studied [6,7], the disorder’s precise etiology and pathophysiology remain elusive.

Genetic mechanisms certainly play an important role, and strong causal effects on ASD have long been known [8] for several rare genetic variants, such as tuberous sclerosis [9] and the fragile X syndrome [10]. Further genetic studies have shown the association of hundreds of distinct gene variants with autism, accounting, however, for only a fraction of ASD diagnoses [8,11]. Only recently, in the largest genome-wide association study (GWAS) for ASD to date, several single loci with genome-wide significance for association with ASD were reported for the first time [12]. Not only the phenotypic expression of ASD but also its etiology appear to be heterogeneous [13,14].

In recent studies, immunological mechanisms have increasingly been discussed as risk factors for altered neuronal development in ASD patients [15]. In particular, there is increasing evidence that maternal immune-dysfunction during gestation may lead to an ASD-like phenotype in susceptible offspring [11,16]. The first hints of the role of maternal prenatal infections were identified in the context of a significant increase in ASD after a rubella epidemic in the US in 1964 [17,18]. Furthermore, studies in large Danish registries found an elevated likelihood of ASD in children whose mothers were hospitalized during pregnancy due to a viral infection [19]. The possible importance of immune-dysfunction for an increased ASD risk is further supported by Scandinavian registry studies that reported a significant association between positive family history for autoimmune diseases and ASD [20,21]. Likewise, children of mothers with systemic lupus erythematosus have a doubled risk for ASD [22]. Potential mechanisms for an accentuated immune activation to affect neurological development are via cytokines, autoantibodies [11], or maternal anti-fetal brain antibodies [23,24]. In line with this, in post-mortem brains and the cerebrospinal fluid (CSF) of ASD patients, a proinflammatory cytokine profile has been found with activated microglia and astrocytes as signs of neuroinflammation [25].

In patients, CSF analysis is the most precise method for investigating subtle inflammation in the central nervous system (CNS) [26,27,28]. Furthermore, there is evidence that CSF circulation, which usually supplies growth hormones and signal molecules and cleanses the brain of harmful substances for proper brain development, is impaired in ASD [29]. Earlier CSF studies in ASD have analyzed children and infants as well as post-mortem material but not adult patients. This may be due to the fact that diagnostic procedures of potential brain disease are initiated at time of diagnosis (i.e., earlier in development and not in adulthood). Another reason might be that no CSF sampling is performed in adult ASD patients who are not characterized by mental retardation or further psychiatric/neurological symptoms. Therefore, this study’s rationale is to retrospectively analyze the CSF findings of adult patients with ASD.

## 2. Participants and Methods

The study was part of a larger project of retrospective biomarker detection that was approved by the local ethics committee (Faculty of Medicine, Freiburg University, 396/18).

### 2.1. Study Sample

We included 36 patients with ASD who had received a lumbar puncture at our tertiary care hospital from 2010 to 2018. The patients were diagnosed by experienced senior physicians following German guidelines [30] as described in detail in earlier papers [31,32] and were classified following ICD-10. Patients with neurodegenerative disorders were excluded from the study; examples include mild cognitive impairment (ICD-10: F06.7) or dementia (ICD-10: F03), substance abuse (ICD-10: F1x.2), or severe neurological diseases other than epilepsy (because of its known association with ASD) [33,34]. The presence of other common comorbid conditions in patients with ASD was not defined as an exclusion criterion; examples include affective disorders (ICD-10: F30-F39), or neurodevelopmental disorders such as attention deficit hyperactivity disorder (ADHD; ICD-10: F90.x), or Tourette syndrome (ICD-10: F95.2).

The control group comprised 39 mentally healthy controls with idiopathic intracranial hypertension (IIH; ICD-10: G93.2), a non-inflammatory neurological disease characterized by increased intracranial pressure of unknown origin. All controls with clearly identifiable secondary forms of intracranial hypertension, those being treated with psychotropic drugs at the time of the lumbar puncture, and those with a history of psychiatric or neurological disorders (except headache) were excluded. This control group was collected in the context of a previous CSF study [35,36,37].

All the clinical and demographic data of the ASD patients were extracted from medical reports. Patients’ psychometric data had been collected as part of routine clinical documentation, including the Clinical Global Impression (CGI) score [38], the Global Assessment of Functioning (GAF) score [4], and psychopathological scores based on the German Association for Methodology and Documentation in Psychiatry (AMDP) [39].

### 2.2. CSF and Instrument-Based Diagnostics

All of the included participants were informed about the lumbar puncture and gave their written informed consent. The CSF analyses were performed in the CSF laboratory at the Department of Neurology (https://www.uniklinik-freiburg.de/neurologie/klinik/diagnostische-einrichtungen/liquor-labor.html) as described in previous studies [40,41,42]. Routine CSF analysis included the determination of white blood cell (WBC) count, total protein concentration, albumin quotient (AQ), immunoglobulin G (IgG) index, and oligoclonal bands (OCBs). Antibodies against cell surface antigens (NMDA-receptor, LGI1, CASPR2, AMPA1/2-receptor, GABA-B-receptor) were identified in the CSF using fixed-cell assays (Euroimmun^®^). Antibodies in serum against paraneoplastic intracellular antigens (Yo, Hu, CV2/CRMP5, Ri, Ma1/2, SOX1, Tr, Zic4, glutamate decarboxylase 65 (GAD65), amphiphysin) were screened using immunoblots (Ravo line assay^®^). In individual cases, the anti-GAD65 antibody levels were measured in serum and CSF additionally using a radioimmunoassay (Medipan^®^, see [28] for comparison). All ASD patients received cerebral magnetic resonance imaging (MRI) assessed by experienced neuroradiologists as well as electroencephalograms (EEGs) assessed by the attending physicians.

### 2.3. Statistical Analyses

Data analysis was conducted with Statistical Package for the Social Sciences, version 25 (IBM Corp., Armonk, NY, USA). Group comparisons of categorical variables were conducted with Pearson’s Chi-squared test. If the result in any of the cells of the contingency table was below 5, Fisher’s exact test was used. One-way analyses of covariance (ANCOVAs) were conducted to determine whether there was a statistically significant difference between the patients and the controls in their CSF basis findings while controlling for the effect of age. Spearman’s rank correlation coefficient was used for correlation analysis between CSF parameters (WBC count, protein concentration, AQ, and IgG index) of the ASD group and their psychometric scores, such as CGI, GAF, and AMDP as well as the number of inpatient stays, suicide attempts, and age. Further ANCOVAs were performed to detect significant influences of gender as well as the presence of abnormalities in EEGs or MRI on CSF parameters of ASD patients while controlling for the effect of age. To control the false discovery rate in multiple testing we calculated adjusted *p*-values with R (R Core Team, 2019) using the Benjamini–Hochberg procedure [43]. Thereby the *p*-values of the CSF basic diagnostics were adjusted together, then the *p*-values of the number of subjects with abnormal CSF diagnostics as a separate group and finally the *p*-values of each correlation of a CSF parameter with the psychometric data. A *p*-value of less than 0.05 was considered statistically significant.

## 3. Results

### 3.1. Clinical and Demographic Data

For clinical and demographic data of the patient and control samples see Table 1. The ASD and the control cohort differed significantly in gender ratio (*p* < 0.001) and age (*p* = 0.03). According to the ICD-10 subgroups of ASD, 34 patients were coded as diagnosed with Asperger syndrome (ICD-10: F84.5) and two with atypical autism (ICD-10: F84.1). All ASD patients had psychiatric comorbidities, of which depression was the most prevalent (75%).

Most of the ASD patients were single (86.1%), lived with their parents/guardians (47.2%) and were either working (30.6%) or in training (30.6%). The majority had achieved intermediate (38.9%) to high (38.9%) education. Most patients had a positive family history for some psychiatric disease in first-degree relatives (63.9%).

### 3.2. CSF Diagnostics

At the time of lumbar puncture, most of the ASD patients (75%) were treated with psychotropic medications, of which the most prevalent were atypical neuroleptics (52.8%) and antidepressants (55.6%). An exact listing of psychotropic medications used at the time of lumbar puncture is provided in Table 2.

Of the 36 ASD patients, three (8.3%) showed a slightly elevated WBC count, 12 (33.3%) an increased total protein, and six (16.7%) an increased age-dependent AQ. CSF-specific OCBs were found in one patient with ASD (2.8%) and in none of the control patients, and no patient showed an increased IgG index. In summary, 16 of the 36 patients (44.4%) presented abnormal CSF basic diagnostic findings, which differed significantly (*p* = 0.013) from respective findings in controls with abnormal CSF measures (7 of 39; 18%). The ANCOVAs conducted with age correction showed a significant difference between ASD patients and controls in total protein (F_(1,73)_ = 6.450, *p* = 0.003) and AQ (F_(1,73)_ = 5.878, *p* = 0.004), but not regarding WBC counts or IgG index. For this analysis, data from 36 ASD patients and 39 controls were evaluated except for the WBC count, for which data from only 35 controls were available. All results of the CSF basic diagnostic are summarized in Table 3 and Table 4.

In a secondary analysis, we additionally investigated the effect of gender and the interaction between age and gender in these ANCOVAs. Here, the group effect for total protein remained significant (F_(1,70)_ = 4.327, *p* = 0.041) and for AQ a tendency could still be observed (F_(1,70)_ = 3.167, *p* = 0.079). We found a significant interaction between age and gender in the ANCOVAs for total protein (F_(1,70)_ = 6.510, *p* = 0.013) as well as AQ (F_(1,70)_ = 5.299, *p* = 0.024), but gender alone had no significant effect on the models for total protein (F_(1,70)_ = 2.073, *p* = 0.154) and AQ (F_(1,70)_ = 1.834, *p* = 0.180). For WBC count and IgG index no significant group effects or interactions were detected.

Most ASD patients additionally received screenings for antineuronal antibodies against cell surface antigens (*n* = 31) or intracellular antigens (*n* = 34) (Table 5). Two of the patients tested were positive for anti-GAD65 antibodies in serum and CSF, and one patient had a nonspecific reaction for anti-Yo antibodies in serum.

### 3.3. Instrument-Based Diagnostics

All patients received an EEG and an MRI scan of the brain. Sixteen ASD patients (44.4%) showed abnormalities in their MRI scans, the most frequent being white matter lesions/cerebral microangiopathy (25%). In the EEGs, generalized intermittent slow activity was found in five patients (13.9%) and focal slowing in three patients (8.3%). One patient (2.8%) had a history of epilepsy and showed corresponding epileptiform discharges with frontal spike wave complexes (Table 6).

### 3.4. Clinical Characteristics of ASD Patients with Anti-GAD65 Antibodies 

The two ASD patients who tested positive for anti-GAD65 antibodies in serum (patient (1): 14 U/mL; patient (2) 101 U/mL; reference <2.0 U/mL) displayed an increased antibody index (patient (1): 50.4, patient (2): 20.3; reference ≤1.5) indicating an intrathecal anti-GAD65 antibody synthesis. Both patients were relatively young, patient (1) was male and patient (2) was female. Both patients suffered from comorbid depression; patient (1) also suffered from tic disorder and patient (2) from anxious jitteriness and Hashimoto thyroiditis. Both patients had no diabetes mellitus (HbA1c levels were in the normal range) and no epilepsy. However, in patient (1), initially a slightly elevated WBC count in the CSF (6/µL; reference <5/µL; with normalization in the follow-up), increased streptolysin antibody levels, as well as anatomical alterations in the form of asymmetric lateral ventricles in the MRI and abnormal frontal slow waves in the EEG (which were partly generalized) were identified. A complementary [^18^F] fluorodeoxyglucose positron emission tomography of the brain showed age-appropriate cerebral glucose utilization without patterns for an acute inflammatory CNS disease. In patient (2), the laboratory results, MRI, EEG, and CSF basis diagnostics were all essentially normal. 

### 3.5. Correlation Analyses

For the ASD group we found significant negative correlations between WBC count and AMDP scores for formal thought disorders (r = −0.336, *p* = 0.045; *n* = 36) and delusion (r = −0.457, *p* = 0.005; *n* = 36) as well as number of suicide attempts (r = −0.405, *p* = 0.014; *n* = 36). This corresponds to moderate effect sizes of the correlation coefficients according to Cohen but were not significant after adjustments that control for the false discovery rate (*p* = 0.27/0.09/0.126). Furthermore, age correlated with total protein (r = 0.338, *p* = 0.043; *n* = 36) and AQ (r = 0.474, *p* = 0.004; *n* = 36). All other correlations between the CSF basic diagnostic parameters and the psychometric data were not significant. No significant influence of gender or the presence of EEG or MRI abnormalities on CSF parameters of ASD patients was detected.

## 4. Discussion

The main findings of this study are higher CSF levels of AQs in patients with ASD indicate potential blood–brain barrier (BBB) dysfunction and an intrathecal synthesis of anti-GAD65 antibodies in two patients with ASD. Overall, we found abnormal CSF basic findings in almost half of the ASD patients—significantly more than in the control group.

### 4.1. Significance of Increased CSF Protein Levels

Altogether the ASD group presented significantly higher CSF levels of total protein (478.1 vs. 309.3 mg/dL) and AQs (5.84 vs. 3.93) compared to the control group. Accordingly, 33.3% of ASD patients had an increased total protein and 16.7% an increased albumin ratio, which compares well to figures in similarly aged patients with schizophreniform psychosis (total protein increase in psychosis in 42.4%, increased AQs in 21.8%) [40] and to the previously reported proportion of neurological patients with a non-inflammatory disease of the CNS with isolated BBB dysfunction (17.5%) [45]. The serum/CSF AQ is clinically considered the “gold standard” for the assessment of BBB function [46]. Since albumin is only produced in the liver, it can reach the CSF only via the BBB. Nevertheless, it is still a matter of debate whether BBB dysfunction as measured with elevated AQs is the sequel of “leakage” at the site of small vessels and capillaries or a reduced CSF drainage in the context of disturbances of CSF flow [47,48]. For ASD, an altered CSF flow was suggested by Shen et al. [29]. In their study of the MRIs of infants who later developed autism, the authors observed an increased extra-axial CSF volume as a possible sign of reduced CSF circulation [49]. Interestingly, the protein concentration in the extra-axial CSF was also significantly elevated compared to healthy children [29]. Thus, it has been suggested that an impaired CSF circulation may lead to an altered distribution of growth factors and signal molecules in the brain as well as a disturbed cleansing of neurotoxins, which can cause neuroinflammation when they accumulate [29].

### 4.2. EEG and MRI Findings

The link between epilepsy and autism is well known, and thus an EEG is an important diagnostic procedure in patients with ASD. However, in addition to the detection of epileptiform discharges in a subgroup of patients, increased nonspecific EEG abnormalities in the form of, for example, slowing or asymmetries [50], as well as hints of long-range underconnectivity [51] in ASD patients have been described. The current study included one patient with comorbid epilepsy and epileptiform discharges, and 19% of the patients presented only focal or generalized slow activity. A previous study with a small sample size discerned a statistically significant difference between ASD patients and healthy controls in the rate of slow activity after hyperventilation [52]. One quarter of ASD patients in the current study showed nonspecific white matter alterations in their MRI. Other mental illnesses, such as depression, present similar rates [41]. It is assumed that these are caused by micro- or macroangiopathic diseases resulting from a dysfunction of the neurovascular unit, which can also lead to a BBB dysfunction [53]. Neuroinflammation can lead to defects in the neovascular unit and, reciprocally, defects in the neurovascular unit can also promote neuroinflammatory processes in the brain. However, the significance and clinical relevance of these white matter alterations remain unclear. Further studies in larger and more homogeneous subgroups of ASD patients are needed to develop a better understanding.

### 4.3. Antibody Findings

The presence of brain-reactive antibodies in patients with ASD has been reported in numerous studies [54]. Accordingly, Singer et al. [55] observed a greater prevalence of autoantibodies in the serum of autistic children than in their non-autistic siblings or healthy controls (in human brain slices of the caudate nucleus, putamen, prefrontal cortex, cerebellum, and cingulate gyrus). Moreover, the presence of brain-reactive antibodies in either the patient or the mother is associated with a more severe degree of autism [56] and has been reported to lead to markedly greater brain enlargement in preschool children who later develop ASD [57]. In the current study, the authors identified two patients (5.6%) who presented an intrathecal synthesis of anti-GAD65 antibodies. GAD65 is an enzyme that catalyzes the intracellular synthesis of the inhibitory neurotransmitter γ-amino butyric acid (GABA) through the decarboxylation of glutamate in brain cells and the pancreatic islet β-cells of the pancreas. Autoantibodies against GAD65 are common at the onset of type I diabetes but have also been reported in various neurological disorders, such as stiff person syndrome, cerebral ataxia, and, in some cases, in epilepsy and limbic encephalitis [58,59]. Interestingly, type I diabetes is a common co-morbidity not only of patients with ASD but also of their mothers and first-degree relatives [54,60,61]. In the healthy population, the prevalence of anti-GAD65 antibodies ranged from 0.5% to 1.1% [62,63]. Small studies in patients with ASD have described a higher incidence in ASD patients, ranging from 5% (three of 60 patients) [64] to 15% (three of 20 patients) [65]. There are, however, contradictory findings that may be related to diverse subgroups of ASD patients or to measurement techniques for anti-GAD65 antibodies [66]. It is important to note that most studies only tested antibodies in serum and did not comment on BBB integrity or the presence of antibodies in the CSF. Presumably, the antibodies are relevant for brain alteration only when they can reach the brain tissue from the serum through a “leaky” BBB due, for instance, to injury or inflammation or when they are synthesized intrathecally [67]. Although why are anti-GAD65 antibodies associated with diabetes mellitus in one patient, stiff person syndrome in another, and with autism in a third? The time at which antibodies reach specific organs as well as genetic predispositions (HLA haplotype) seem to play important roles [68], and environmental factors may be important. The pathophysiology of neuropsychiatric disease in association with anti-GAD65 antibodies is poorly understood to date. The gene expression of GAD65 appears to be reduced in ASD patients in certain brain regions, such as the cerebellar dentate nuclei [69,70], and also in the cortex of mice whose mothers during pregnancy received the anti-epileptic drug valproate, which is associated with a high risk of autism [71]. Moreover, hypermethylation of the *GAD1* promoter has been observed in the offspring of mice that received a nonspecific immune response by means of a poly (I:C) injection during pregnancy [72]. One possible pathomechanism is the direct intracellular binding of anti-GAD65 antibodies to the cytoplasmic GAD65 through unknown mechanisms leading to a change in GABAergic transmission. While a strong binding has been demonstrated in vitro [73], the application of anti-GAD65 antibodies in the CSF of mice in vivo did not lead to a change in hippocampal GABAergic transmission [74]. Thus, the pathophysiological functioning of anti-GAD65 antibodies seems to be more complex than, for example, in the case of the immunoglobulin G antibodies against the surface N-methyl D-aspartate receptor. In this context, we need to consider that GAD65 antibodies themselves may not be pathophysiologically active, but rather an immunological epiphenomenon of an unknown underlying pathomechanism [75]. Thus, the formation of anti-GAD65 antibodies could be secondary to the destruction of GABAergic neurons by, for instance, hitherto unidentified antibodies and the release of intracellular GAD into CSF [75,76]. This would also explain the clinical heterogeneity of diseases associated with anti-GAD65 antibodies and their different responsiveness to immunosuppressive therapies. In all cases, anti-GAD65 antibodies function at least as a disease marker of an unknown but most likely immune mediated process [76].

Case reports have described severe neurological diseases in association with anti-GAD65 antibodies, such as limbic encephalitis and epilepsy, that barely responded to classical immunotherapy, such as steroids or intravenous immunoglobulins (IVIGs), and required more aggressive treatment with monoclonal antibodies, such as basiliximab or rituximab [77,78]. Nevertheless, it has been reported at least in one patient with ASD with clinically relevant high anti-GAD65 antibodies and comorbid type I diabetes, that there was a relevant benefit from therapy with IVIGs [64].

### 4.4. Limitations

This study was performed openly and retrospectively. The decision to conduct lumbar punctures followed clinical criteria to exclude a secondary organic pathology and was not a routine procedure; therefore, the patient group is not representative of all patients with ASD. In addition, the control group’s unclear IIH pathophysiology may have introduced a confounder to data in this group. In patients with IIH, larger amounts of CSF were removed due to therapy. Given that a clear reduction of the AQ between the first 4 mL and the last 4 mL of a total of 24 mL of CSF of a lumbar puncture has been described [79,80], false low values in the control group are possible. Moreover, the AQ may vary due to body weight, smoking, degenerative disc disease, hypothyroidism, or gender [80,81]. Particularly noteworthy is the gender difference between our patient and control groups. However, we did not find a gender effect in our secondary analysis of the CSF parameters but in the gender corrected ANOVA, however, only the group difference in the protein concentrations was still significant, whereas the AQ showed only a tendential increase in the ASD patients. Our findings could therefore be partly caused by a gender difference. Furthermore, the measured values for total protein and sometimes additionally AQ of one third of the ASD patients were also increased compared to established reference values. Differences on other CSF parameters like WBC and IgG due to gender have not been observed.

Psychotropic medication may also have had an influence on the total protein in the CSF and AQs. While three quarters of the ASD patients were medicated, the control group did not receive psychiatric medication. It has been indicated that, for example, antipsychotic drugs in bipolar patients may lead to an increased AQ [82]. However, other studies found no influence of antipsychotic [83,84,85] or antidepressant [86] medication on CSF properties. Finally, depression and other comorbidities may have confounded our results.

## 5. Conclusions

The present retrospective study on CSF measures provides no clear-cut positive evidence for relevant inflammatory alterations in adult ASD, however, it does suggest a trend toward a BBB dysfunction in some adult patients with ASD. The identification of anti-GAD65 antibodies in some patients with ASD may be relevant for future research in an effort to define etiological and clinical sub-phenotypes of ASD informing more personalized treatment approaches.

## Figures and Tables

**Table 1:** Clinical and demographic data of patients and controls.

	Patients with Autism (*n* = 36)	Controls (*n* = 39)	Statistics
**Gender**	13 F:23 M	33 F:6 M	*p* < 0.001
**Average age at time of lumbar puncture (age range)**	28.94 ± 9.9 (18–53 years)	34.6 ±12.0 (18–61 years)	*p* = 0.03
**Diagnosis**			
F84.5	*n* = 34 (94.4%)	-	
F84.1	*n* = 2 (5.6%)	-	
G93.2	-	*n* = 39 (100%)	
**Neuropsychiatric comorbidity**			
Depression	*n* = 27 (86.1%)	-	
ADHD	*n* = 6 (16.7%)	-	
Schizophreniform disorders	*n* = 5 (13.9%)	-	
History of epilepsy	*n* = 2 (5.6%)	-	
Obsessive compulsive disorder	*n* = 2 (5.6%)	-	
Personality disorder	*n* = 2 (5.6%)	-	
Others	*n* = 7 * (19.4%)	-	
**Civil status**			
Single	*n* = 31 (86.1%)		
Married	*n* = 3 (8.3%)		
Unknown	*n* = 2 (5.6%)		
**Educational level**			
Low	*n* = 5 (13.9%)		
Intermediate	*n* = 14 (38.9%)		
High	*n* = 14 (38.9%)		
Unknown	*n* = 3 (8.3%)		
**Employment**			
Unemployed	*n* = 7 (19.4%)		
Working	*n* = 11 (30.6%)		
Education/training	*n* = 11 (30.6%)		
Occupational disability	*n* = 4 (11.1%)		
Others/unknown	*n* = 3 (8.3%)		
**Living situation**			
Alone	*n* = 13 (36.1%)		
With partner/family	*n* = 2 (5.6%)		
With parents/custodian	*n* = 17 (47.2%)		
Psychiatric transitional arrangement	*n* = 1 (2.8%)		
Others/unknown	*n* = 3 (8.3%)		
Family history for any psychiatric disease **			
Positive	*n* = 23 (63.9%)		
Negative	*n* = 9 (25.0%)		
Unknown	*n* = 4 (11.1%)		
**Number of previous inpatient stays**			
None	*n* = 12 (33.3%)		
1	*n* = 4 (11.1%)		
2	*n* = 7 (19.4%)		
3	*n* = 3 (8.3%)		
More than 3	*n* = 7 (19.4%)		
Unknown	*n* = 3 (8.3%)		
**Burden of acute events**			
None	*n* = 6 (16.7%)		
Mild	*n* = 17 (47.2%)		
Intermediate	*n* = 5 (13.9%)		
Severe	*n* = 4 (11.1%)		
Extreme	*n* = 1 (2.8%)		
Unknown	*n* = 3 (8.3%)		
**Burden of long-term life circumstances**			
None	*n* = 2 (5.6%)		
Mild	*n* = 10 (27.8%)		
Intermediate	*n* = 9 (25.0%)		
Severe	*n* = 9 (25.0%)		
Extreme	*n* = 2 (5.6%)		
Unknown	*n* = 4 (11.1%)		
**Number of suicide attempts**			
None	*n* = 28 (77.8%)		
1	*n* = 6 (16.7%)		
2	*n* = 1 (2.8%)		
Unknown	*n* = 1 (2.8%)		

* Bipolar disorder, anorexia nervosa, narcolepsy, Tourette syndrome, insomnia, substance induced psychotic disorder, dissociative disorder. ** In first-degree relatives. Abbreviations: F = female, M = male, ADHD = attention deficit hyperactivity disorder, *n* = number.

**Table 2:** Psychotropic medication at time of lumbar puncture.

	Patients with ASD (*n* = 36)
**Class of Medication**	
Selective serotonin reuptake inhibitor	*n* = 6 (16.7%)
Selective serotonin/noradrenaline reuptake inhibitor	*n* = 4 (11.1%)
Tricyclic antidepressants	*n* = 6 (16.7%)
Bupropion	*n* = 3 (8.3%)
Mirtazapine	*n* = 2 (5.6%)
Typical neuroleptics	*n* = 1 (2.8%)
Atypical neuroleptics	*n* = 19 (52.8%)
Lithium	*n* = 1 (2.8%)
Anticonvulsants	*n* = 7 (19.4%)
Benzodiazepine	*n* = 1 (2.8%)
Methylphenidate	*n* = 2 (5.6%)
Melatonin	*n* = 5 (13.9%)
Others	*n* = 4 * (11.1%)
**Number of Different Medication Classes per Patient**	
Same class/only one drug	*n* = 9 (25.0%)
Two drugs	*n* = 7 (19.4%)
Three drugs	*n* = 9 (25.0%)
More than three	*n* = 2 (5.6%)
Unmedicated	*n* = 9 (25.0%)

The number refers to different drug classes. If several drugs of the same class were taken, only one was included. * One patient with comorbid ADHD received atypical off-label treatment with levodopa + carbidopa (this was changed to bupropion after lumbar puncture); another patient took biperiden and clonidine. Abbreviations: ASD = Autism spectrum disorder; ADHD = attention deficit hyperactivity disorder; *n* = number.

**Table 3:** CSF basis diagnostics.

	Reference [44]	ASD Patients (*n* = 36)	Controls (*n* = 39)	Statistics (Unadjusted *p*-Value)
WBC count (Mean ± SD)	<5/µL	1.94 ± 1.37	2.60 ± 7.59 *	*p* = 0.698 (0.698)
Total protein (Mean ± SD)	≤450 mg/L	478.14 ± 391.1	309.33 ± 142.5	***p* = 0.009 (0.003)**
Albumin quotient (Mean ± SD)	<40 years: 6.5 × 10^−3^; 40−60 years: 8 × 10^−3^; >60 years: 9.3 × 10^−3^	5.84 ± 5.66	3.93 ± 1.81	***p* = 0.009 (0.004)**
IgG index (Mean ± SD)	≤0.7 mg/L	0.50 ± 0.39	0.50 ± 0.038	*p* = 0.257 (0.193)

The control group was created for an earlier project and the results were published earlier [35,36,37]. Abbreviations: CSF = cerebrospinal fluid; WBC = white blood cell, SD = standard deviation, IgG = immunoglobulin G, *n* = number. * Data of only 35 controls are available. One of them suffered from pleocytosis (46 cells/µL), which normalized independently, and was interpreted as reactive pleocytosis. *p*-Values adjusted by Benjamini–Hochberg procedure. Bold = significant at *p* ≤ 0.05.

**Table 4:** Number of subjects with abnormal CSF diagnostics.

	ASD Patients (*n* = 36)	Controls (*n* = 39)	Statistics (Unadjusted *p*-Value)
Increased WBC count (≥5/µL)	*n* = 3 (8.3%) *	*n* = 1 (2.9%) **	*p* = 0.115 (0.086)
Increased total protein (>450 mg/L)	*n* = 12 (33.3%)	*n* = 6 (15.4%)	*p* = 0.115 (0.069)
Increased age-dependent albumin quotient (<40 years: 6.5 × 10^−3^; 40−60 years: 8 × 10^−3^; >60 years: 9.3 × 10^−3^)	*n* = 6 (16.7%)	*n* = 2 (5.1%)	*p* = 0.191 (0.143)
Increased IgG index (>0.7 mg/L)	*n* = 0	*n* = 0	-
CSF specific OCBs	*n* = 1 (2.8%) ***	*n* = 0 ****	*p* = 0.324 (0.324)
Patients with abnormal CSF basis diagnostics	16/36 (44.4%)	7/39 (18%)	*p* = 0.013

The control group was created for an earlier project and the results previously published (see [35,36,37]). The number of patients with abnormalities in CSF diagnostics, as a summary of previous tests, was not corrected for multiple testing. Abbreviations: WBC = white blood cell; SD = standard deviation; IgG = immunoglobulin G; OCBs = oligoclonal bands; *n* = number. * 5, 6 and 6 white blood cells without blood admixture. ** Only data of 35 controls are available. One of them suffered from reactive pleocytosis (46 cells/µL), which regressed independently to normal WBC counts and was interpreted as reactive pleocytosis. *** Two additional findings were not considered positive: one patient had an isolated OCB in the CSF, another patient had 1–2 very weak bands in the CSF. **** Only available from 38 (of 39) control patients. *p*-Values adjusted by Benjamini–Hochberg procedure. Bold = significant at *p* ≤ 0.05.

**Table 5:** Antineuronal autoantibody findings.

Patients with ASD (*n* = 36)	Antibodies against Cell Surface Antigens in CSF	Antibodies against Paraneoplastic Intracellular Antigens in Serum
Not analyzed	*n* = 5 (13.9%)	*n* = 2 (5.6%)
Negative	*n* = 31 (86.1%)	*n* = 32 (88.2%)
Positive	*n* = 0	*n* = 2 * (5.6%)

* Two patients displayed anti-glutamate decarboxylase 65 (GAD65) antibodies, additionally one patient had a non-specific reaction for anti-Yo antibodies, which was not rated as a positive result. Abbreviations: ASD = Autism spectrum disorder, CSF = cerebrospinal fluid; *n* = number.

**Table 6:** MRI and EEG pathologies in the ASD patient group.

MRI Abnormalities	Patients (*n* = 36)
White matter lesions/cerebral microangiopathy	*n* = 12 (33.3%)
Generalized cortical atrophy	*n* = 2 (5.6%)
Localized cortical atrophy	*n* = 1 (2.8%)
Pineal cyst	*n* = 4 (11.1%)
Other anatomical variants	*n* = 4 * (11.1%)
More than one abnormality	*n* = 7 (19.4%)
Normal findings	*n* = 20 (55.6%)
**Total number of MRI abnormalities**	16/36 patients (44.4%)
**EEG pathologies**	**Patients (*n* = 36)**
Continuous generalized/regional slow activity	-
Intermittent generalized slow activity	*n* = 5 (13.9%)
Intermittent focal slow activity	*n* = 3 (8.3%)
Epileptiform discharges	*n* = 1 (2.8%)
Absence of EEG pathologies	*n* = 28 (77.8%)
**Total number of EEG pathologies**	8/36 patients (22.2%)

* Arachnoidal cyst, asymmetric lateral ventricles, asymmetric vertebral artery, enlarged Virchow–Robin’s space. Abbreviations: MRI = magnetic resonance imaging; EEG = electroencephalogram; *n* = number.

## Abbreviations

ADHD	Attention deficit hyperactivity disorder
AMDP	Association for Methodology and Documentation in Psychiatry (in German: Arbeitsgemeinschaft für Methodik und Dokumentation in der Psychiatrie)
ANCOVA	One-way analysis of covariance
AQ	Albumin quotient
ASD	Autism spectrum disorder
BBB	Blood–brain barrier
CGI	Clinical Global Impression
CNS	Central nervous system
CSF	Cerebrospinal fluid
EEG	Electroencephalography
F	Female
GABA	γ-amino butyric acid
GAD65	Glutamate decarboxylase 65
GAF	Global Assessment of Functioning
GWAS	Genome-wide association study
IgG	Immunoglobulin G
IIH	Idiopathic intracranial hypertension
IVIG	Intravenous immunoglobulins
M	Male
MRI	Magnetic resonance imaging
*n*	Number
OCB	Oligoclonal band
SD	Standard deviation
WBC	White blood count
y	Years
